# *Telipogon diabolicus* (Orchidaceae, Oncidiinae), a new species from southern Colombia

**DOI:** 10.3897/phytokeys.65.8674

**Published:** 2016-07-08

**Authors:** Marta Kolanowska, Dariusz L. Szlachetko, Ramiro Medina Trejo

**Affiliations:** 1 Department of Plant Taxonomy and Nature Conservation, The University of Gdańsk, Wita Stwosza 59, 80-308 Gdańsk, Poland; 2 Department of Biodiversity Research, Global Change Research Institute ASCR, Bělidla 4a. 603 00 Brno, Czech Republic; 3 Sibundoy Valley, Alto Putumayo, Colombia

**Keywords:** Andean orchid, biodiversity, new species, Putumayo

## Abstract

A new species of the orchid genus *Telipogon*, *Telipogon diabolicus*, is described and illustrated. The information about its habitat is provided. The novelty resembles *Telipogon tabanensis* and *Telipogon guacamayensis* and it is characterized by the translucent flowers, the glabrous, distinctly clawed petals, the transversely elliptic lip, and the gynostemium ornamented with long setose hairs on both sides and shorter hairs at the apex.

## Introduction

The Neotropical genus *Telipogon* was established about 200 years ago by Karl Kunth (1816) who recognized only two species within newly published taxon: *Telipogon angustifolius* and *Telipogon latifolius*. The former orchid was earlier recognized as *Tradescantia nervosa* and transferred to *Telipogon* by Druce (1917). During the 19th century, over 40 new species within the genus were described by Reichenbach (e.g. Reichenbach 1854, 1877). Pfitzer (1887) included *Telipogon* in Notylieae tribe while Schlechter (1915) proposed to unite *Trichoceros*, *Telipogon* and *Stellilabium* in a separated subtribe named Telipogoninae. Dressler and Dodson (1960) classified those genera in *Ornithocephalus* alliance, but Schlechter’s proposal was accepted by subsequent morphological taxonomists (e.g. Burns-Balogh and Funk 1986, Dressler 1993, Szlachetko 1995). The results of molecular studies provoked Chase et al. (2003, 2015) to lump all genera mentioned before together with over 50 other taxa in Oncidiinae.

Until 2005 about 190 specific epithets were applied to *Telipogon*. Williams et al. (2005) revealed that, according to the results of phylogenetic studies, *Stellilabium* is embedded within *Telipogon* and 36 species of the former genus were transferred by the authors to *Telipogon*. The novelties within the genus have been frequently published in the last years (e.g. Dressler 2007, Nauray Huari and Galán de Mera 2008, Baquero and Fortunato 2012, Jiménez Pérez 2012, Collantes and Martel 2015) and so far a total of about 250 specific epithets are listed under *Telipogon* according to The International Plant Names Index (2016).

In the most recent catalogue of Colombian plants (Bernal et al. 2015) almost 3600 orchid species representing nearly 250 genera are included. However, there is no doubt that hundreds of species occurring in this country remain undiscovered. Only in 2015 over 20 novelties were published based on material collected in Colombia (e.g. Kolanowska and Szlachetko 2015, Rodríguez Martínez and Blanco 2015, Szlachetko and Kolanowska 2015, Vieira-Uribe and Jost 2015). During the recent studies on Colombian orchids a distinctive species of *Telipogon* was found and it is described here as new species.

## Description of the new species

### Telipogon diabolicus

Taxon classificationPlantaeAsparagalesOrchidaceae

Kolan., Szlach. & Medina Tr. sp. nov.

urn:lsid:ipni.org:names:77155897-1

Figs 1 , 2

#### Diagnosis.

Species similar to *Telipogon tabanensis* and *Telipogon guacamayensis*, distinguished by the translucent, relatively small flowers with sepals reaching 9-10 mm in length, transversely elliptic lip and prominently clawed petals.

#### Type.

COLOMBIA. Putumayo/Nariño: Páramo de Bordoncillo, 3180 m, 7 Nov 2015, *R. Medina et al. S15/13* (Holotype JAUM!; Isotype JAUM!; UGDA-DLSz! - drawing).

#### Description.

Stem 5.5–9 cm tall, stem abbreviated. Leaves 2–4.5 × 0.4–1.3 cm, conduplicate, relatively fleshy, ovate-lanceolate to oblanceolate, attenuate towards the base, subacute. Inflorescence 6–9 cm long, 2–3-flowered, peduncle triquetrous. Flowers simultaneous, tepals translucent with reddish veins, gynostemium and lip callus dark violet-maroon. Floral bracts 7–9 mm long, cucullate, ovate, acute. Pedicel and ovary 15–20 mm long, triquetros. Sepals similar, keeled on the back side. Dorsal sepal 9–9.5 × 4–4.5 mm, concave, ovate-elliptic, acute, 3-veined. Lateral sepals 9–10 × 3–4 mm, concave, ovate-elliptic, acute, somewhat oblique, 3-veined. Petals 10–12 × 9–9.3 mm, rhombic in outline, broadly elliptic ovate to transversely elliptic above prominent claw, acuminate, 9-veined, claw basally thickened and densely ciliolate with papillate margins. Lip 9–9.3 mm long, 10–11 mm wide, transversely elliptic, acute at the apex, 15-veined, margins glandular-ciliate, basal margins with short spines; callus 3–4 mm × 2.5–3 mm, ovate-cordate, densely ciliate with several setae spread all over its surface. Gynostemium about 3 mm tall, clinandrium 3-lobed, lateral bundles of setose hairs elongate up to 3 mm long, the dorsal bundle covering the anther much shorter, area around the stigma papillate, with several setae. Capsule 15–20 mm long.

**Figure 1.**
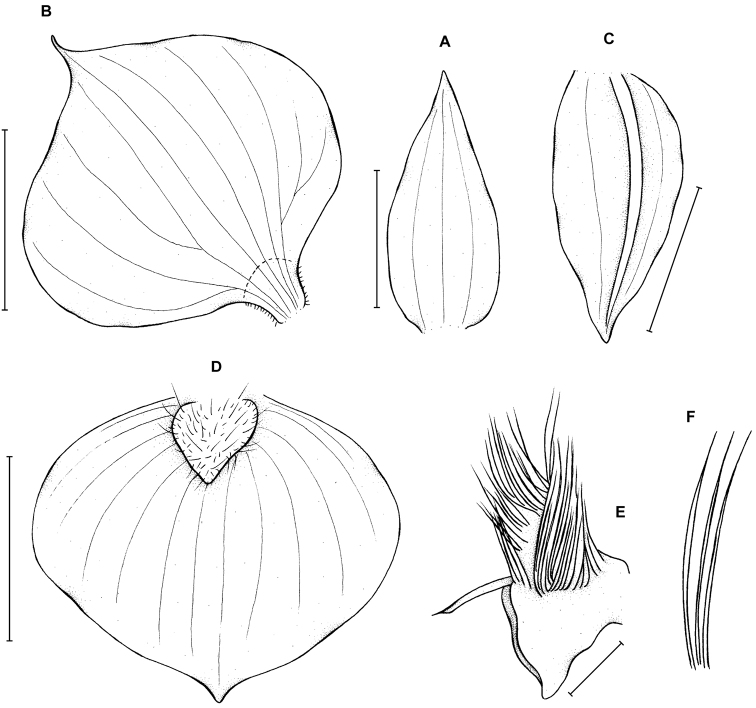
*Telipogon diabolicus* Kolan., Szlach. & Medina Tr. **A** Dorsal sepal **B** Petal **C** Lateral sepal **D** Lip **E** Gynostemium **F** Setae of the gynostemium. Drawn by N. Olędrzyńska from the holotype. Scale bars: **A–D** = 5 mm, **E** = 2 mm.

**Figure 2.**
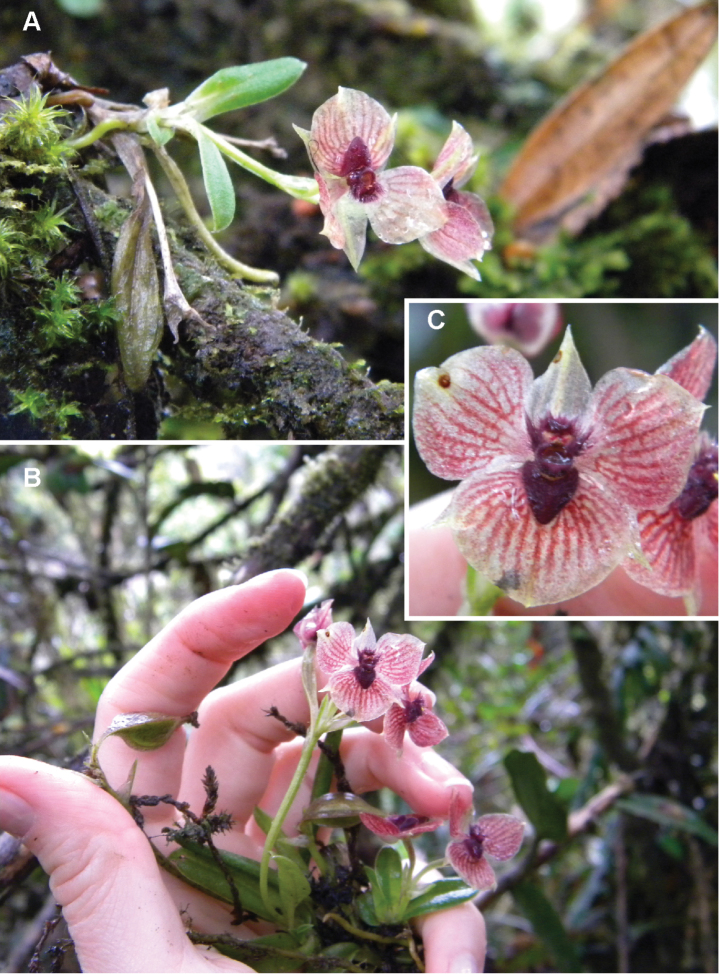
*Telipogon diabolicus* Kolan., Szlach. & Medina Tr. **A–B** Plant habit **C** Flower closeup. Photos by M. Kolanowska.

#### Etymology.

The specific name refers to the distinctive gynostemium which resembles devil’s head.

#### Distribution and ecology.

So far this species is known exclusively from southern Colombia, on the border between departments Putumayo and Nariño. It was found growing epiphytically in wet, dwarf montane forest at the edge of páramo. The population which was observed during the field study consists of about 30 specimens of which only several were adult, flowering plants.

#### Conservation status.

IUCN Red List category: Critically Endangered, [CR B2ab(iii)]. This species is only known from the type specimens, which represent one location in highly vulnerable habitat near the main road Pasto-Mocoa. It is expected that the current reconstruction of this road will have negative impact on the habitat of *Telipogon diabolicus*.

#### Discussion.

**Figure 3.**
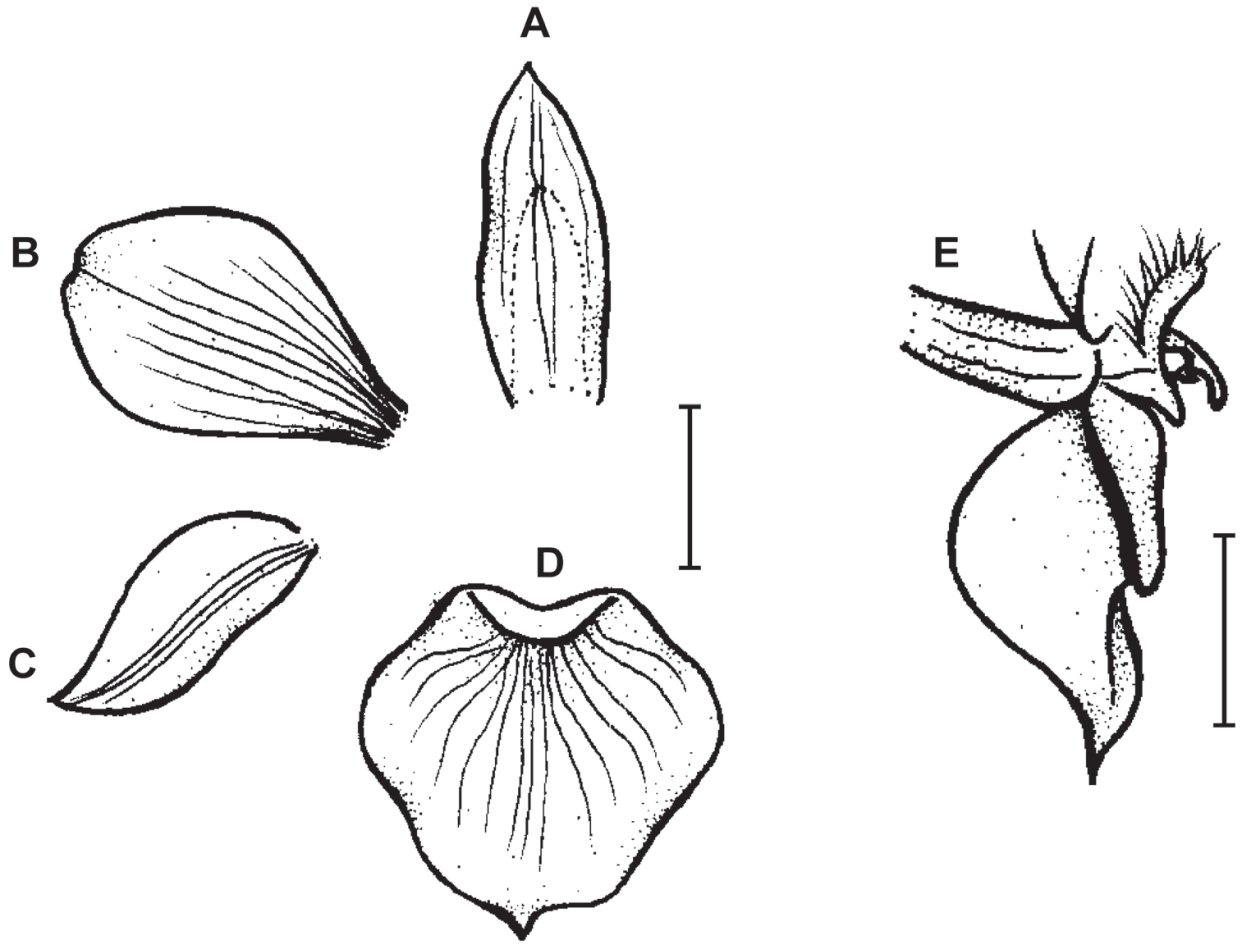
*Telipogon guacamayensis* Dodson & R. Escobar. **A** Dorsal sepal **B** Petal **C**: Lateral sepal **D** Lip **E** Lip, side view. Redrawn by N. Olędrzyńska from original illustration presented by Dodson and Escobar (*in* Dodson 1989a). Scale bars: 5 mm.

**Figure 4.**
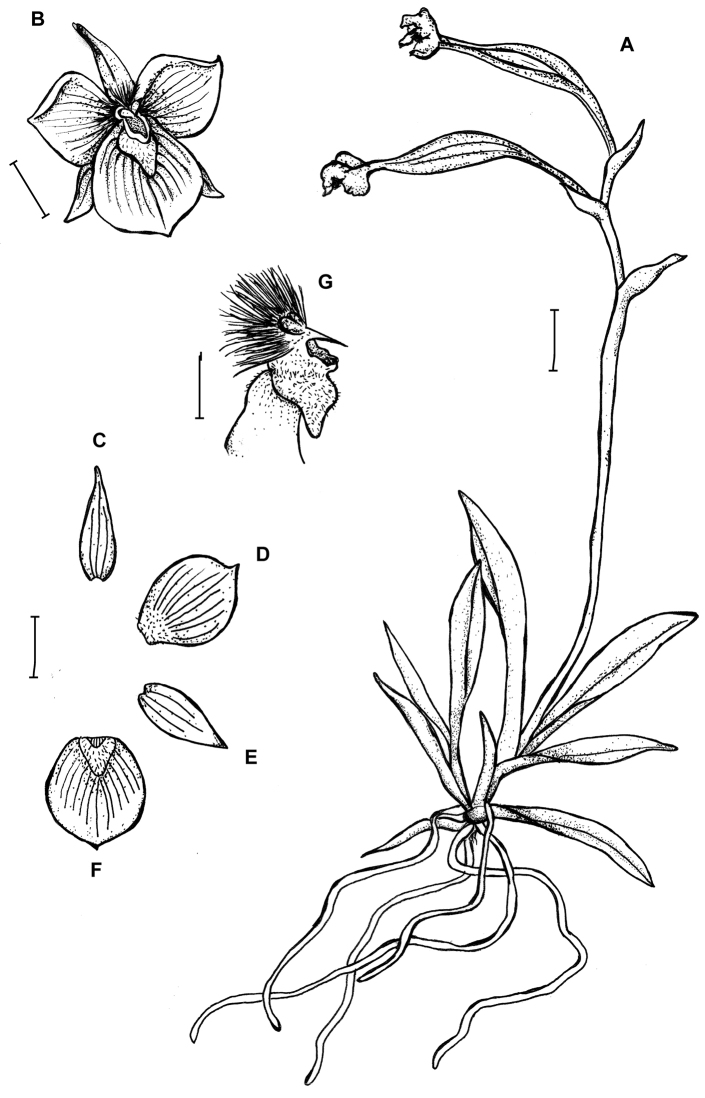
*Telipogon tabanensis* Dodson & R. Escobar **A** Habit **B** Flower **C** Dorsal sepal **D** Petal **E** Lateral sepal **F** Lip **G** Gynostemium and lip callus, side view. Redrawn by M. Staroń from original illustration presented by Dodson and Escobar (1993). Scale bars: **A–F** = 10 mm, **G** = 5 mm.

The new species can be misidentified with its Colombian congener *Telipogon tabanensis* Dodson & R. Escobar (1993) and Ecuadorian *Telipogon guacamayensis* Dodson & R. Escobar (*in* Dodson 1989a), but both those orchids are characterized by yellow flowers with dark (wine-red to maroon) gynostemium and callus (vs flowers translucent in *Telipogon diabolicus*). Flowers of both *Telipogon tabanensis* and *Telipogon diabolicus* are resupinate (non-resupinate in *Telipogon guacamayensis*), but those of *Telipogon tabanensis* are much larger – sepals are about 17 mm long (vs 9–10 mm in *Telipogon diabolicus*), petals reach 20 mm in length (vs 12 mm). Petals of the former are densely spinose-hirsute at the base while in the new species and in *Telipogon guacamayensis* (Fig. 3) they are glabrous. In both *Telipogon tabanensis* (Fig. 4) and *Telipogon guacamayensis* the lip is 17-veined (vs 15-veined in *Telipogon diabolicus*) and it is subtrullate (*Telipogon guacamayensis*) or elliptic (*Telipogon tabanensis*). All three species are characterized by presence of prominent, more or less cordate basal lip callus which is about 6 mm long in *Telipogon tabanensis* and *Telipogon guacamayensis* (up to 4 mm in *Telipogon diabolicus*). Only in *Telipogon diabolicus* the basal lip margin is covered with short spines. The additional difference between *Telipogon tabanensis* and the new species is found in the gynostemium ornamentation. In the former orchid it is covered with equally long setose hairs while in *Telipogon diabolicus* (and *Telipogon guacamayensis*) the lateral bundles of hairs are elongated, longer than the dorsal bundle covering the anther.

**Figure 5.**
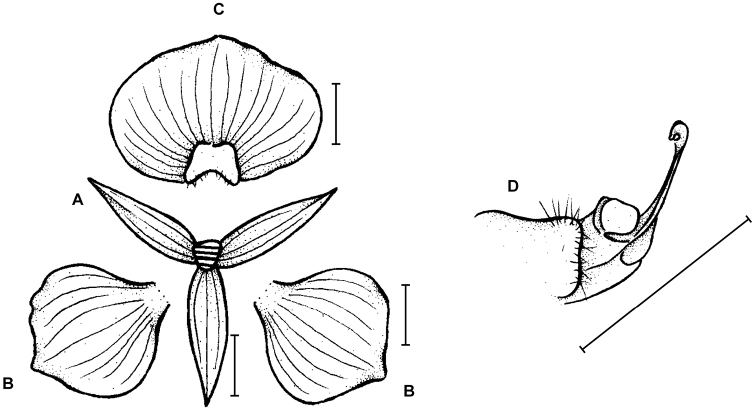
*Telipogon intis* Braas **A** Sepals **B** Petal **C** Lip **D** Gynostemium. Redrawn by N. Olędrzyńska from Dodson and Bennett (*in* Dodson 1989). Scale bars: 10 mm.

**Figure 6.**
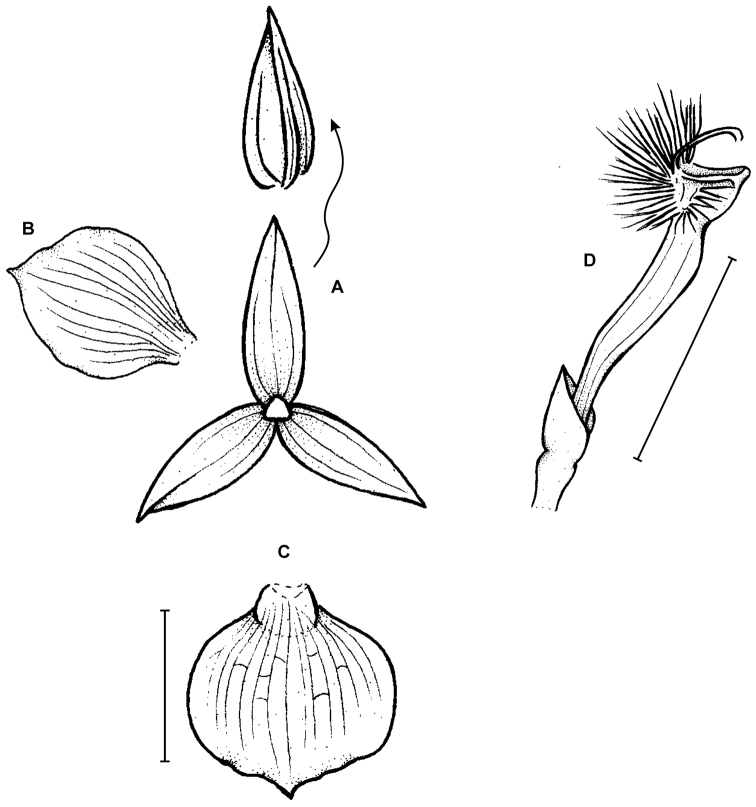
*Telipogon lueri* Dodson & Bennett **A** Sepals **B** Petal **C** Lip **D** Gynostemium. Redrawn by N. Olędrzyńska from original illustration presented by Dodson and Bennett (*in* Dodson 1989b). Scale bars: 20 mm.

**Figure 7.**
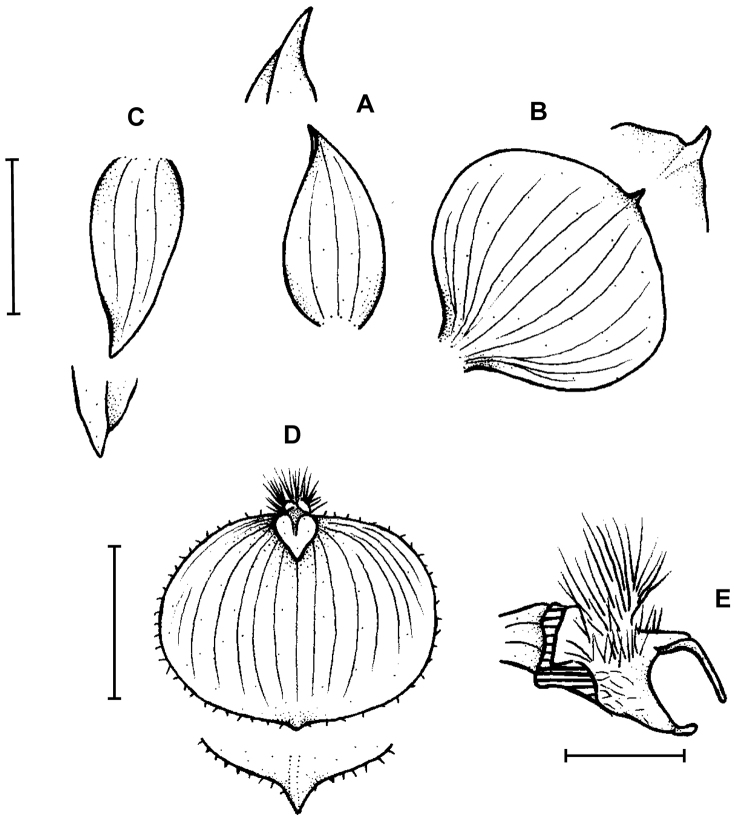
*Telipogon mendiolae* Dodson & Bennet.t **A** Dorsal sepal **B** Petal **C** Lateral sepal **D** Lip **E** Gynostemium. Redrawn by N. Olędrzyńska from original illustration presented by Dodson and Bennett (*in* Dodson 1989b). Scale bars: **A–D** = 10 mm, **E** = 3 mm.

The most distinguishing character of the new species are prominently clawed petals. At the best of our knowledge, this character is not found in any other Colombian species of the genus. Interestingly, at least 3 species from Peru share this feature, i.e. *Telipogon intis* Braas (Fig. 5), *Telipogon lueri* Dodson & Bennett (Fig. 6) and *Telipogon mendiolae* Dodson & Bennett (Fig. 7). In the first of the Peruvian species aforementioned the obtuse lip has 17 nerves, petals are acute and gynostemium is sparsely setose on clinandrium. *Telipogon mendiolae* can be characterized by transversely elliptic, obtuse lip with 17 nerves, and transversely elliptic, shortly apiculate petals. Flowers of this species are about twice larger than those of *Telipogon diabolicus*. *Telipogon lueri* differs from our new species by having twice larger flowers, densely setose gynostemium and petals with 11 nerves.

**Figure 8.**
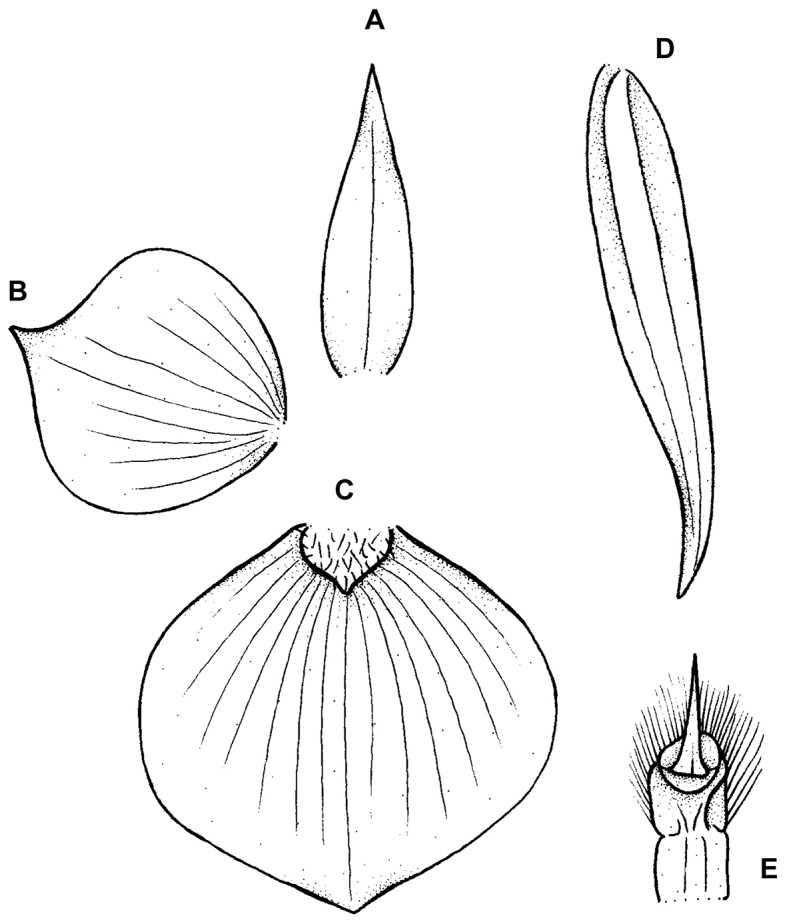
*Telipogon ecuadorensis* Schltr. **A** Dorsal sepal **B** Petal **C** Lateral sepal **D** Lip **E** Gynostemium. Redrawn by N. Olędrzyńska from Schlechter (1929).

**Figure 9.**
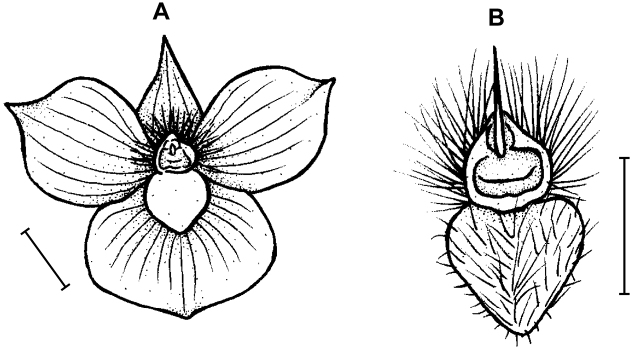
*Telipogon bruchmuelleri* Rchb.f **A** Flower **B** Gynostemium and lip callus. Redrawn by N. Olędrzyńska from Dodson and Dodson (*in* Dodson 1984). Scale bars: 5 mm.

*Telipogon diabolicus* somewhat resembles also Ecuadorian *Telipogon ecuadorensis* Schltr. (Fig. 9) and *Telipogon bruchmuelleri* Rchb.f. (Fig. 8) known from Ecuador and Venezuela. In all aforementioned species the lip is similar in form, i.e. more or less transversely elliptic with ovate-cordate basal callus. Unlike in *Telipogon diabolicus* the gynostemium of *Telipogon bruchmuelleri* and *Telipogon ecuadorensis* is densely covered by setose hairs (vs setose hairs found only on clinandrium), and petals are sessile (vs clawed).

### Key to identification of *Telipogon diabolicus* and similar species

**Table:** 

1	Petals distinctly clawed	**2**
–	Petals subsessile	**5**
2	Gynostemium almost glabrous, very sparsely setose exclusively on clinandrium	***Telipogon intis***
–	Gynostemium densely covered by hairs	**3**
3	Lip ecallose	**4**
–	Lip with prominent, ovate-cordate callus	***Telipogon diabolicus***
4	Petals transversely elliptic	***Telipogon mendiolae***
–	Petals ovate	***Telipogon lueri***
5	Petals densely spinose-hirsute at the base	***Telipogon tabanensis***
–	Petals glabrous	**6**
6	Petals 5- or 7-veined	***Telipogon bruchmuelleri***
–	Petals 9-veined	**7**
7	Lip 13 × 12 mm, 17-veined	***Telipogon guacamayensis***
–	Lip 23 × 26 mm, 15-veined	***Telipogon ecuadorensis***

## Supplementary Material

XML Treatment for Telipogon diabolicus
